# Histamine in Diabetic Cardiovascular Complications

**DOI:** 10.31083/RCM47801

**Published:** 2026-05-06

**Authors:** Shanshan Hu, Gaofeng Zeng, Yao Tang, Qun Wang, Jianfeng Wu, Xiangdong Yang

**Affiliations:** ^1^Clinical Medicine Research Center of Arteriosclerotic Disease of Hunan Province, The Second Affiliated Hospital, Hengyang Medical School, University of South China, 421002 Hengyang, Hunan, China; ^2^Shanghai Institute of Cardiovascular Diseases, Zhongshan Hospital, Fudan University, 200032 Shanghai, China

**Keywords:** diabetic cardiomyopathy, histamine, H_1_R

## Abstract

Diabetic cardiovascular complications are the primary cause of diabetes-associated mortality. The pathogenesis of diabetic cardiomyopathy is complex; the main clinical manifestations include inflammation, hyperinsulinemia, mitochondrial dysfunction, endoplasmic reticulum stress, and coronary microcirculation disorders. Among these factors, inflammatory responses play a pivotal role in diabetic cardiomyopathy. The accumulation of histamine secreted by macrophages in multiple tissues of patients with diabetes mellitus is crucial for the onset and progression of the disease, particularly diabetic cardiovascular complications. Histamine and associated receptor-mediated signaling pathways are implicated in diabetic cardiovascular complications; however, the specific mechanisms remain unclear and warrant further investigation.

## 1. Introduction

By 2045, nearly 693 million people worldwide are expected to have diabetes. Diabetic complications are the most common cause of mortality among patients with diabetes. Cardiovascular diseases are dominant complications of diabetes, among which heart failure is the most prevalent, with an incidence of approximately 19–26% in patients with diabetes [1, 2, 3]. According to the Framingham study, patients with diabetes have a 5–6 times higher risk of developing cardiovascular disease than those without diabetes [4, 5]. Further, Murtaza *et al*. [6] reported that the risk for heart failure in patients with diabetes increases by 8% for every 1% increase in the baseline level of glycosylated hemoglobin A1c (HbA1c) in the absence of hypertension, obesity, and coronary artery disease [7]. Therefore, diabetes status seriously affects cardiovascular function.

Recent research has revealed that histamine plays a key role in diabetes and its associated complications. Histamine is a biologically active monoamine. In diabetes, various cytokines are released, including histamine, which contributes to obesity, glucose tolerance, and insulin resistance [8]. Histamine plays a crucial role in the cardiovascular system, exerting vasoactive, chronotropic, inotropic, and cardiac rhythm responses. The mechanism of histamine in diabetic cardiovascular complications remains unclear, and gaining a deeper understanding of the role of histamine in diabetes and its cardiovascular implications is of clinical importance.

## 2. Literature Review

### 2.1 Diabetic Cardiovascular Complications

In 1972, Rubler and colleagues [9] discovered a new type of cardiomyopathy, termed diabetic cardiomyopathy (DCM), through autopsies of four patients with diabetes who had died of heart failure. These patients lacked cardiac diseases, such as coronary artery disease, hypertension, and valvular heart disease, but showed pathological features including diffuse myocardial fibrosis, hypertrophy, and microvascular smooth muscle cytosis [10, 11]. In the initial stage, heart failure manifests as normal systolic function and impaired diastolic cardiac function, eventually developing into heart failure with preserved ejection fraction. Heart failure is characterized by coronary microvascular endothelial dysfunction and reduced ejection fraction owing to myocardial death [12, 13]. Hyperglycemia and insulin resistance lead to pathological reactions, including oxidative stress, mitochondrial dysfunction, endothelial dysfunction, endoplasmic reticulum stress, and abnormal intracellular calcium ion levels within cardiomyocytes, which impair glucose and lipid metabolism [14]. It leads to cardiac remodeling and impaired cardiac function owing to excessive triglyceride accumulation within cardiomyocytes, extracellular protein deposition, and increased production of advanced glycation end products (AGEs) [15, 16].

Chronic low-grade inflammation is a key characteristic of diabetic cardiomyopathy, contributing to its onset and progression [17]. Bone marrow-derived CD11b^+^ macrophages are primary inflammatory cells found in patients with diabetic cardiomyopathy [18]. The accumulation of pro-inflammatory macrophages and alterations in the function of insulin target cells synergistically induce insulin resistance, leading to hyperglycemia [19, 20]. Under conditions of hyperglycemia, macromolecular substances—such as proteins, amino acids, lipids, and nucleic acids—undergo condensation, rearrangement, cleavage, and oxidative modification with aldehyde groups, without enzymatic hydrolysis, to form AGEs, which are pro-oxidative metabolic derivatives [21, 22].

AGEs activate the innate immune response by binding to the AGE receptor on various innate immune cells, including macrophages, natural killer (NK) cells, dendritic cells (DCs), neutrophils, and epithelial cells, which regulate the adaptive immune response [23]. AGEs induce the migration and release of inflammatory factors within macrophages, including tumor necrosis factor-α and interleukin-1β, through the advanced glycosylation end-product specific receptor (AGER)–nuclear factor kappa-b (NF-κB) signaling pathway [24]. It induces glycocalyx shedding of endothelial cells, promoting the expression and exposure of endothelial cell adhesion molecules and increasing endothelial permeability [25, 26, 27]. Endothelial cell adhesion molecules recruited white blood cells, which triggered the inflammatory response and generated various inflammatory factors [28]. Subsequently, c-Jun N-terminal kinase (JNK)-dependent caspases are activated, leading to cardiomyocyte apoptosis, excessive cardiomyocyte remodeling, permanent fibrosis, and cardiac stiffness [29, 30, 31, 32]. In addition, hyperglycemia can activate the renin-angiotensin-aldosterone system (RAAS) and mineralocorticoid receptor signaling pathway, further contributing to ventricular remodeling in diabetic cardiomyopathy [33]. In summary, the inflammatory and oxidative responses induced by hyperglycemia, hyperlipidemia, and insulin resistance lead to myocardial cell apoptosis and necrosis, resulting in myocardial remodeling and contractile dysfunction in diabetes mellitus.

### 2.2 Histamine and Its Receptors

**Fig. 1.**
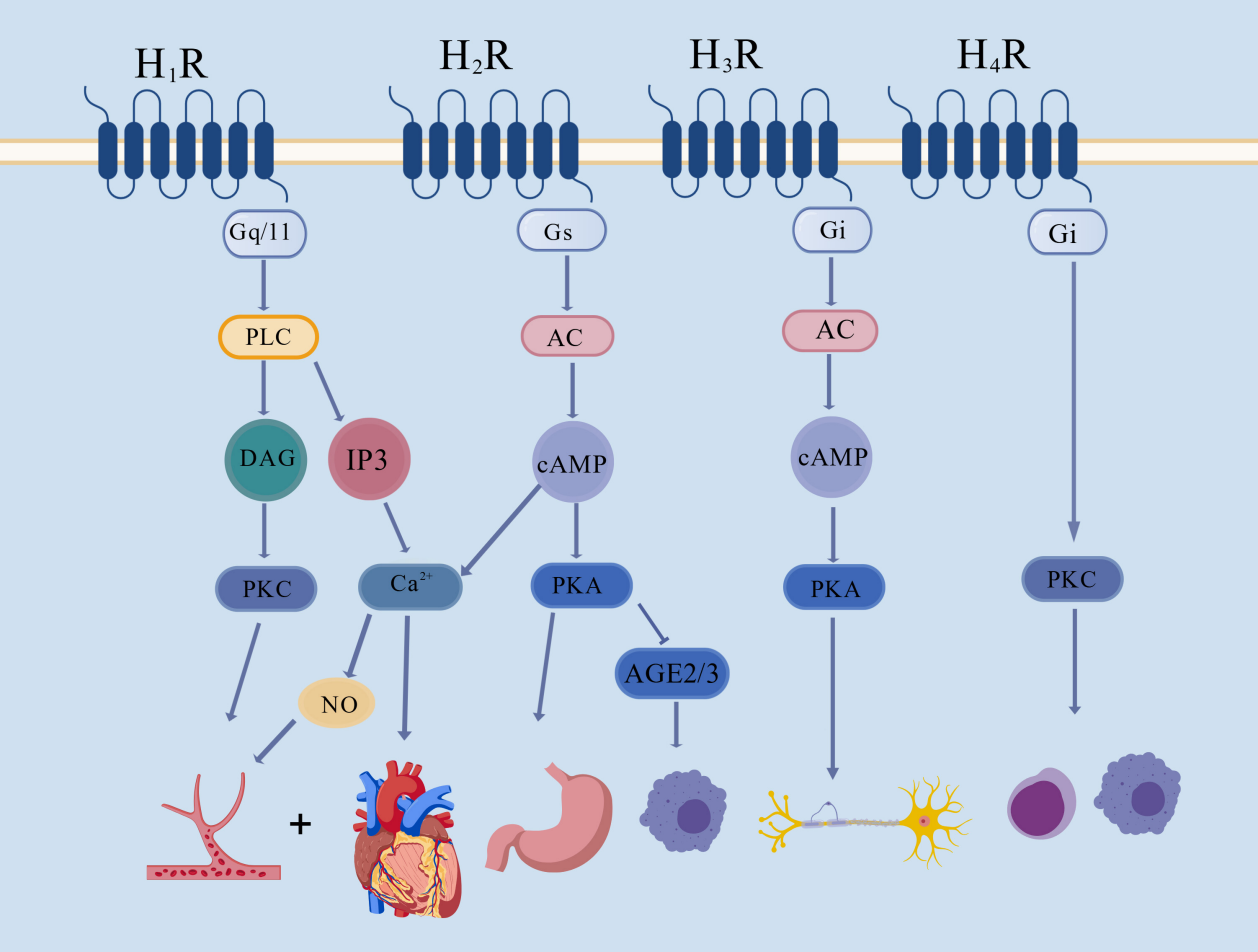
**Summary of the mechanisms underlying the H_1_R-H_4_R signaling pathway**. Histamine regulates the cardiovascular system by stimulating the H_1_R–PLC–DAG–PKC and the H_1_R–PLC–IP_3_–Ca^2+^ signaling pathways. Histamine enhances gastric acid secretion after stimulating the H_2_R–AC/cAMP signaling pathway. The activation of H_3_R affects the central nervous system by stimulating the AC–PKA signaling pathway. Histamine may trigger an immunoreaction via the H_4_R–PKC signaling pathway [58]. H_1_R, histamine H1 receptor; H_2_R, histamine H2 receptor; H_3_R, histamine H3 receptor; H_4_R, histamine H4 receptor; Gq/11, G protein-coupled-receptor family q/11; Gs, G protein-coupled-receptor family s; Gi, G-protein-coupled-receptor family i; PLC, phospholipase C; DAG, diacylglycerol; PKC, proteinase C; IP_3_, inositol triphosphate; AC, adenylate cyclase; cAMP, cyclic adenosine monophosphate; PKA, protein kinase A; NO, nitric oxide; AGE2/3, advanced glycosylation end-product receptor 2/3. This drawing platform is a web-based platform. The company name is as follows: Hong Kong. Coloring Technology (Hong Kong) Co., Limited.

In the early 20th century, histamine was first discovered as a small molecular amine with vasodilator properties [34]. Histamine can be derived *in vitro* and *in vivo*, and endogenous histamine is transformed from L-histidine. Exogenous histidine obtained from ingested food is synthesized into histamine through three main pathways. First, L-histidine can be produced from urocanic acid by histidine lyase [35, 36]. Cis-urocanic acid can alleviate edema and erythema resulting from subacute inflammation and protect the skin against ultraviolet damage [37]. Second, L-histidine and β-alanine combine to form carnosine through the action of carnosine synthetase, which regulates pH and antioxidation [38]. Third, L-histidine is converted into histamine by histidine decarboxylase (HDC) and catabolized through cyclic methylation and oxidative deamination by diamine oxidase [39, 40, 41]. The extracellular deamination of histamine is catalyzed by diamine oxidase, and its intracellular methylation is catalyzed by histamine-N-methyltransferase (HNMT) [42]. Histamine is primarily released by mast cells, neutrophils, gastrointestinal chromaffin cells, and DCs, among other cell types. Histamine regulates various cellular functions by activating histamine receptors [43, 44]. Histamine receptors are transmembrane G protein-coupled receptors, and each receptor subtype has a distinct G protein, comprising histamine H1 receptor (H_1_R), histamine H2 receptor (H_2_R), histamine H3 receptor (H_3_R), and histamine H4 receptor (H_4_R) [45, 46, 47]. Specifically, the H_1_R, H_2_R, H_3_R, and H_4_R bind to Gq, Gs, and Gi [48], and the expression of H_1_R, H_2_R, and H_3_R is increased in the hypothalamus [49, 50]. Histamine is involved in mediating physiological and pathological processes, such as allergic reactions, gastric acid secretion, and nerve transmission through these receptors [51, 52, 53]. The H_1_R is expressed in various cell types, including nerve cells, respiratory epithelial cells, endothelial cells, hepatocytes, vascular smooth muscle cells, DCs, and lymphocytes. Upon binding to H_1_R, histamine activates inositol phospholipid hydrolysis and intracellular Ca^2+^ mobilization, thereby participating in diverse cellular processes [54, 55, 56]. In 1940, Dale *et al*. [57] discovered histamine and its receptors in the heart. Although histamine and its receptors have demonstrated involvement in cardiovascular diseases, the specific mechanism of action remains unclear (Fig. 1, Ref. [58]).

### 2.3 Histamine Receptor Antagonists

Histamine is indispensable to the body. Histamine antagonists which primarily include the competitive and inverse agonists to histamine-receptors are equally significant. The histamine antagonists have experienced three innovations. In 1937, the first generation of histamine antagonists was discovered and applied clinically in 1942. The first generation of histamine antagonists included chlorpheniramine, diphenhydramine, promethazine [59]. However, the clinical application of histamine antagonists was associated with a series of adverse reactions, such as hallucinations, fainting, and insomnia. Histamine antagonists combine with muscarinic receptors, serotonin receptors, and adrenaline receptors and pass through the blood-brain barrier, triggering severe central reactions [60]. Hence, first-generation antihistamines are clinically used to treat allergies. Second-generation histamine antagonists, such as cetirizine, desloratadine, fexofenadine, and clarityne, are widely used in clinical settings to treat acute urticaria and rhinitis [61]. Through selective binding to H_1_R, second-generation histamine antagonists are more effective and safe than first-generation histamine antagonists. In addition, these antagonists do not pass through the blood-brain barrier, and they reduce the risk of some adverse reactions, such as sedation and dryness.

### 2.4 Histamine Induces Vascular Dilation in Diabetes

It has been postulated whether HDC-histamine-H_1_R signaling is implicated in diabetic cardiovascular complications. Some studies have revealed that histidine and its metabolites are involved in type 2 diabetes [62, 63, 64]. Cavalher-Machado and colleagues discovered that in the aortic endothelial cells of streptozotocin (STZ)-induced diabetic mice, the histamine content was increased by 138%, HDC activity was elevated by 250%, and histaminase activity was reduced by 50% compared with those in control mice [65, 66, 67]. Solís *et al*. [68] found that H_1_R expression in the telencephalon of diabetic rats was 1.7 times higher than that in control rats, which may be related to neural differentiation in diabetes. Histamine is a vasoactive monoamine that can activate H_1_R on endothelial cells to induce endothelium-dependent vasodilation by triggering H_1_R-independent vasodilation and H_2_R endothelial-independent vasoconstriction [69]. Histamine stimulates Ca^2+^ release from the vascular endothelium by activating H_1_R and promoting the synthesis of nitric oxide (NO) for the endothelial nitric oxide synthase (eNOS) system in vascular endothelial cells, further relaxing blood vessels [70, 71, 72]. Hyperpolarization of calcium-activated K^+^ channels increases the permeability of vascular tissue and exacerbates leakage from diabetic vessels [73, 74, 75]. The activation of H_2_R downregulates AGE-2 and AGE-3-induced adhesion molecule expression, cytokine production, and lymphocyte proliferation through the cyclic adenosine monophosphate (cAMP)–protein kinase A (PKA) pathway [76, 77, 78].

Hyperglycemia is one of the causes of cardiovascular diseases in diabetes. Benter *et al*. [79] and Badavi *et al*. [80] found that a high concentration of glucose attenuates histamine-induced vascular dilation, leading to glucosamine formation in the digestive tract of diabetes. Following H_1_R blockade, hyperglycemia triggered Ca^2+^ release from endothelial cells, reduced NO synthesis in vascular endothelial cells, and weakened vasodilation [81, 82, 83]. In addition, the by-products of hyperglycemia (AGEs) inhibit histamine-induced vasodilation, promote histamine secretion from macrophages, and trigger chymotrypsin release to activate the RAAS, consequently causing irreversible cardiac damage [81]. In diabetic rats, HDC activity increased with shear stress, experimental hypertension, and dietary hypercholesterolemia [84]. Moreover, histamine deficiency can lead to aggravated cardiac and renal impairment in cardiorenal syndrome, decreased left ventricular fractional shortening, reduced glomerular filtration rate, and increased urinary protein secretion [85]. When histamine receptors are blocked, the cardiovascular function of patients with diabetes is affected. Cimetidine, an H_2_R antagonist, suppressed the expression of endothelial adhesion factors and P-selectin in neutrophilic granulocytes induced by hyperglycemia in diabetes [86]. Pini *et al*. [87] discovered that H_4_R antagonists attenuate the progression of diabetic nephropathy by inhibiting kidney fibrosis and inflammation. In conclusion, histamine induces vascular dilation by activating its receptor signaling pathways in diabetes; nonetheless, the specific underlying mechanisms remain unclear.

### 2.5 Impact of Histamine on Cardiac Rhythm and Atrioventricular (AV) Conduction in Diabetes

In recent years, some studies have revealed that histamine modulates the arrhythmic processes of the heart [88, 89]. Cardiac dysfunction and arrhythmias were improved in rats treated with H_1_R antagonists, which induced early reperfusion [90]. The myocardium of patients with diabetes undergoes complex mechanical and electrical changes, which mainly manifest as prolongation of the Q-T interval and increased QTC dispersion; these changes are closely related to the incidence of sudden death in patients with diabetic cardiomyopathy [91]. Histamine can change the electrical rhythm of the heart. High concentrations of histamine stimulated somatic nerves when locally injected into the femoral artery and caused hypotension, bradycardia, tachypnea, and hyperpnea [92]. In rats, histamine induced transient slowing of the heart rate followed by tachycardia, and it gradually weakened and then increased myocardial contractility. Histamine exerted a rapid transient negative inotropic effect beginning at 30 nM and very significantly at 1 mM, followed by a sustained positive inotropic effect in the WT rat [93]. Activation of H_1_R could induce a positive inotropic effect on the heart, while blockade of H_1_R reduced myocardial ejection fraction [94]. Therefore, the application of histamine antagonists can also affect electrocardiac conduction. First-generation histamine receptor antagonists have anti-muscarinic and anti-α-adrenergic receptor effects; they induce a dose-dependent prolongation of the Q-T interval and trigger tachycardia [95]. Second-generation H_1_R antagonists, such as astemizole and terfenadine, can block the rectified K^+^ current and prolong the monophasic action potential, Q-T interval, and polymorphic ventricular arrhythmia [96]. Under conditions of insufficient endogenous histamine in diabetic mice, the action potential repolarized and was maximally prolonged [91]. Histamine and its signaling pathways regulate cardiac electrical signals in diabetic cardiomyopathy.

### 2.6 Myocardial Contractility in Diabetic Cardiomyopathy

According to reports, histamine can affect the contractile function of the myocardium. Previous study has revealed that mast cells infiltration and histamine content increased in the myocardial tissue of patients with chronic heart failure; further, intracellular cAMP content and myocardial contractility were increased by histamine receptor activation [97]. Watkins *et al*. [98] found that injection of histamine enhanced myocardial fractional shortening and the velocity of myocardial fiber shortening, thereby augmenting the contractility of the left ventricular myocardium in mice [98, 99]. Histamine briefly enhances and then restrains myocardial contractility in mice [92]. In addition, histamine stimulates the renin-angiotensin system and enhances catecholamine secretion, which strengthens myocardial contractility [100]. Considering that histamine can enhance myocardial contractility, histamine receptor antagonists may also influence myocardial contractility. Watkins *et al*. [98] demonstrated that the cimetidine significantly reduced histamine-induced fractional shortening and the velocity of cardiac fiber shortening of mice. Propyl benzyl chloride, a H_3_R agonist, activated the sympathetic nervous and renin-angiotensin systems and promoted vasopressin secretion. These effects resulted in a dose-dependent increase in systolic blood pressure and enhanced survival rates in mice subjected to hemorrhagic shock [101]. Thus, histamine can enhance myocardial contractility in diabetic mice.

### 2.7 Histamine Regulates Energy Metabolism in Diabetic Myocardium

Histamine is involved in regulating the body’s energy homeostasis. Histamine can regulate energy expenditure, energy intake, circadian rhythms, and temperature through related receptors in the hypothalamus. In the central nervous system, histamine regulates leptin through H_1_R and mediates the expression of uncoupling protein and glucose metabolism [102]. Histamine regulates lipid and glucose metabolism in the liver and skeletal muscle via the H_2_R-mediated adiponectin system in peripheral tissues [103]. Stimulating H_1_R in the hypothalamus suppresses appetite, whereas H_1_R antagonists are strongly associated with increased food intake, weight gain, and obesity [49]. H_1_R activates various key proteins involved in insulin receptor intracellular signaling, increases glycogen synthesis, and causes weight gain [104]. In addition, the interaction of Semicarbazide-sensitive amine oxidase (SSAO), including histamine, with vanadate significantly stimulates tyrosine phosphorylation of insulin receptor substrate (IRS)-1 and IRS-3 and tyrosine protein kinase or inhibits protein tyrosines. Some signalling molecules, such as H_2_O_2_ in the combination of SSAO and vanadate, cause glucose transporter 4 (GLUT4) recruitment to the cell surface and the stimulation of glucose transport in adipose cells [105, 106]. When endogenous histamine is lacking, mice develop impaired glucose tolerance and are prone to hyperglycemia and autoimmune diabetes. Thus, histamine receptor antagonists affect energy metabolism. The H_1_R antagonist astemizole can alter the genes involved in intracellular calcium homeostasis, regulate the intracellular calcium content, and induce intracellular glycogenolysis [107, 108, 109]. Cimetidine reduces appetite and body weight and improves the glucose levels, insulin sensitivity, and lipid profile of patients with type 2 diabetes [110]. However, Thangam *et al*. [111] reported obesity, hyperinsulinemia, and hyperleptinemia in mice with H_3_R knockdown. Applying H_3_R antagonists increased insulin secretion and reduced body, inflammatory response, oxidative stress, hyperglycemia, and hyperlipidemia [112]. Collectively, there are different opinions on the role of histamine and its receptor signaling pathways in regulating energy metabolism; in general, it is agreed that histamine can promote energy metabolism.

## 3. Limitations 

Although the existing experimental results have demonstrated that histamine and its signaling pathways play significant roles in cardiovascular complications of diabetes. There are still limitations. Studies about histamine and its signaling pathways were still insufficient in diabetic cardiomyopathy and cardiovascular complications. Secondly, the research articles and data related to histamine and its signaling pathways are not sufficiently comprehensive, and the analysis of the relevant arguments is not thorough enough in diabetic cardiomyopathy and cardiovascular complications. We will comprehensively explore the research results and conduct further in-depth research in the future.

## 4. Conclusions

**Fig. 2.**
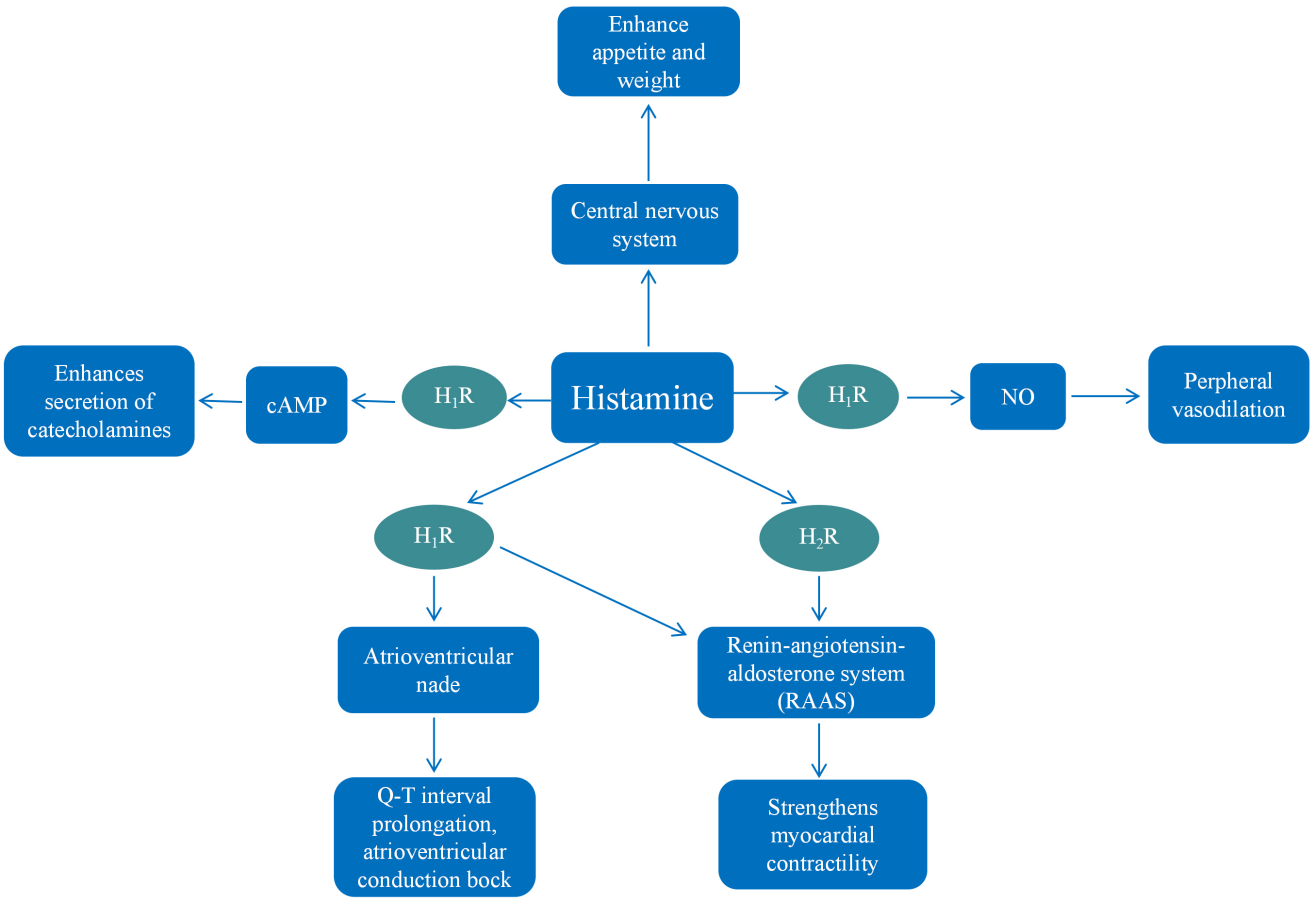
**Summary of the mechanisms of histamine in diabetic cardiomyopathy**. Histamine promotes the production and release of NO in vascular endothelial cells, causing dilated peripheral vasodilation. Histamine enhances the secretion of catecholamines and strengthens myocardial contractility by activating cyclic adenosine monophosphate (cAMP) and the renin-angiotensin-aldosterone system (RAAS). Histamine prolongs the Q-T interval and blocks atrioventricular conduction by stimulating the atrioventricular node. Besides, histamine enhances energy absorption, appetite, and weight gain by acting on the central nervous system.

Histamine is involved in the development of vascular complications in diabetes—a topic that remains understudied and is subject to controversy. Histamine regulates blood glucose levels and diabetes pathogenesis [113, 114], modulates blood vessel contraction and relaxation in diabetes, and regulates the electrocardiogram signal transduction, cardiac contractility, and energy metabolism of myocardial tissue. Histamine triggers peripheral vasodilation by promoting the production and release of NO in vascular endothelial cells, enhances the secretion of catecholamines and strengthens myocardial contractility by activating cAMP and the RAAS, and prolongs the Q-T interval and blocks atrioventricular conduction by stimulating the atrioventricular node. Moreover, histamine increases energy absorption, appetite, and weight gain by acting on the central nervous system (Fig. 2). In mammalian myocardial tissue, histamine exhibits positive chronotropic effects, negative dromotropic effects, positive inotropic effects, and increased cardiac automaticity.
